# How is China’s energy security affected by exogenous shocks? Evidence of China–US trade dispute and COVID-19 pandemic

**DOI:** 10.1007/s43937-021-00002-6

**Published:** 2021-07-28

**Authors:** Shi Qiang Liu, Xin Huang, Xiangong Li, Mahmoud Masoud, Sai-Ho Chung, Yong Yin

**Affiliations:** 1 School of Economics and Management, Fuzhou University, Fuzhou, 350108 China; 2 School of Mines, China University of Mining and Technology, Xuzhou, 221116 China; 3 Centre for Accident Research and Road Safety - Queensland, Queensland University of Technology, Brisbane, QLD 4059 Australia; 4 Department of Industrial and Systems Engineering, Hong Kong Polytechnic University, Hong Kong, China; 5 Graduate School of Business, Doshisha University, Kyoto, 602-8580 Japan

**Keywords:** China–US trade dispute, Energy security strategies, Renewable energy transition, Energy policy, Energy patterns

## Abstract

The trade dispute between China and the United States (US) since 2018 and the global COVID-19 pandemic since 2020 has significantly impacted China’s economic development. As China’s energy sources heavily depend on imports, its economic viability is becoming more and more risky. This study proposes a novel conceptual framework, involving macroeconomic, industrial and geopolitical factors, to evaluate China’s energy security as a major player in the trade dispute. This study also provides a comprehensive strategy for policymakers to make better decisions on reforming renewable energy patterns to guarantee energy security and achieve geopolitical advantages. The PESTEL (political, economic, social, technical, environmental and legislative) and SWOT (strengths, weaknesses, opportunities and threats) analytical methods are applied to evaluate the factors and attributes of China’s energy development and energy security in the current background. The China-US bipartite game reciprocity model and the QSPM (Quantitative Strategic Planning Matrix) analysis are conducted to assess which energy security strategy and policy are more suitable to deal with China-US trade dispute. To enhance energy security, China should diversify its energy supply chain, develop new sources of energy supply, advance the shale gas technology, popularise cleaner power-generation plants, increase nuclear-energy safety, introduce energy-conservation measures, promote alternative-energy vehicles, engage in international energy diplomacy, and rebuild international energy transaction and settlement systems.

## Introduction

Over the last two decades, China’s energy needs have skyrocketed because of industrialization and urbanization. China has become the largest importer of crude oil, natural gas and coal worldwide since 2018. Certainly, China is the No. 1 energy consumer in the world [1]. However, China’s total energy supply has been unable to meet true energy demand. In recent years, China’s rapid economic growth has produced the unprecedented energy vulnerability that could threaten its economic sustainability, social stability, and the foundation for its rising aspirations. Obviously, securing energy supplies is crucial to maintain China’s economic growth, social peace, and political stability [2].

Energy is the lifeblood of the modern economy. Energy demand is closely associated with industrial development and consequent urbanization. In fact, the manufacturing industry is the largest energy-consuming sector. Since China took part in economic globalization with the deepening of the reform and opening-up, its coastal region has transformed into an economic heart of export-oriented manufacturing. Especially since being a member of the World Trade Organization (WTO) in 2001, China rose rapidly in the middle and bottom of the global value chain, taking most of the final assembly lines of manufacturing. The world energy consumption centre has shifted to China by the transfer of many industrial workshops from the West into China. Clearly, the distribution of energy consuming centres has already overlapped with that of the manufacturing industry hubs in large cities such as Beijing, Shanghai, Guangzhou, Shenzhen, and Chongqing. Overall, China’s economic structure and energy supply are highly dependent on international trade, while the supply chain system is fully integrated into the dollar-oil cycle [3]. Consequently, global commodity and energy prices have surged in the last two decades. In China, many export-oriented industries are labour-intensive and energy-intensive. Urbanization plays a key role in energy consumption. The urban industrial population has higher energy requirements than the agricultural population. Since the majority of people have moved from rural areas to cities, China’s energy demands have risen sharply. Evidently, electricity consumption is the primary energy consumption in the manufacturing industry. Industrial motors mainly drive China’s electricity consumption growth [4]. For example, China’s electric motors account for over 1/5 of worldwide electricity demand growth [5]. China has built over 100 million kilowatts of new power plants in the last 8 years. In the near future, it is expected that China’s level of electrification in end-use sectors will surge, and the electric energy ratio will increase, reaching 50% by 2030 [6]. In the mid-2020s, China’s industrial energy demand will reach its peak after tripling over the last two decades [7]. In addition, China’s primary energy consumption pattern will change as coal demand will fall from 60.4% in 2017 to 46.3% in 2030, whereas natural gas demand will increase from 6.6 to 13.2% [8]. As China’s major economic mode has been evolving into the third industry or the service sector, it should make sure a more sustainable supply of cleaner and safer energy.

The current trade dispute between China and the United States (US) indicates that their relationship has been changed from complementary to competitive. A trade dispute broke out when China’s “Made in China 2025” plan was released, because the West worried about that China’s growing strength is endangering the international trade status and hegemony of the US [9]. Trade interests and intellectual property rights do not stand alone in the clash between China and the West. As the US and China are competing for hegemony, the trade dispute may last longer than expected [10]. Specifically, China would lose more because non-tariff barriers in the trade dispute will intensify its underlying negative effects. A long-term trade dispute will impede China’s globalization, particularly harming its trading system; and decouple its political, economic and cultural ties, such as cargo shipments, capital flows, technology transfer, international students, and immigration [11].

Energy security is mainly about ensuring the national supply of energy. The long-term security of the energy economy is critical to meet citizens’ needs for end-use energy services [12]. When the trade dispute will progress to a certain extent, energy resources are not solely determined by economic factors. Because China’s economy heavily depends on crude oil and natural gas imports, the China-US decoupling will cause the restriction of China’s balance of payments and energy imports. Specifically, China’s strategical-level crude oil reserves are less than 60 days, far below the threshold of a 180-day security reserve. Although China has the largest oil storage tank cluster in the world, it is still impossible to maintain a sustainable energy supply, particularly in an emergency. Undoubtedly, China will need plenty of low-carbon energy to sustain its economic growth in the coming decades. Under the background of the China-US trade dispute and COVID-19 pandemic, China’s energy needs will probably decrease but the vulnerability of energy supply will increase at the same time. Therefore, China should initiatively reform energy patterns and seek more energy security space to hold out in (and after) the trade dispute and the COVID-19 pandemic.

The contributions of this paper are highlighted as follows. In this study, we analyze China’s energy security from an interdisciplinary but not a solitary-discipline perspective. We are pioneering to explore the context of China’s energy security from the US’s tariff barriers and political confrontation, geopolitics and energy superpower, industrialization and urbanization, foreign exchange reserve security, shale oil technology, etc. Based on characteristics analysis of the explored context, the transformation of energy patterns on Chinese governmental agenda is summarized. Subsequently, we develop qualitative and quantitative analysis methods based on PESTEL (political, economic, social, technical, environmental and legislative), SWOT (strengths, weaknesses, opportunities and threats), and QSPM (Quantitative Strategic Planning Matrix) to assess the implications of China’s energy security strategies and policies. Finally, we propose insightful suggestions on the reform of energy policies and patterns.

## Literature review

In this section, a comprehensive and up-to-date literature review on energy security factors is presented in a specific classification.

### Definition of energy security

Energy security is a multi-faceted concept with economic, political, environmental, social, and technical implications. Basically, an extensive energy security framework includes availability, affordability, accessibility, and acceptability [13]. The concept of energy security is contextual and dynamic, encompassing multiple dimensions like energy affordability and environmental sustainability [14]. Energy security research should transfer from a solitary-discipline topic to an inter-disciplinary research area with a combination of more magnitudes, like energy efficiency, energy policies, energy patterns, energy crisis, energy prices, economic globalization, accessibility of energy services, climate change, mitigation of greenhouse gas emissions, sustainability, and geopolitics [15]. Finally, a systematic theory of energy security involves both logical speculation and system analysis, as well as consideration of both short-term and long-term interests.

### Energy vulnerability and risk management

The energy security concept covers all potential risks with a significant impact on the vulnerability of vital energy systems [16]. Specifically, energy security is concerned about the risks caused by the energy supply chain, ranging from avoiding supply disruptions to mitigating the sudden economic, environmental, and political impacts on the global oil and gas trade. The energy security assessment is a type of risk management problem by referring to the low vulnerability of Vital Energy Systems (VES) [17]. Furthermore, energy vulnerability is regarded as a combination of exposure to risks and resilience to risks [18]. In a sense, some researchers described different types of energy risks in a qualitative way while some researchers believe that quantitative indicators are critical for understanding energy risks [19]. A three-dimensional event-related stress definition (i.e., availability, affordability, and acceptability) describes how an energy system responds when it experiences unexpected events [20]. Traditional energy security theories emphasize reliable and affordable supplies of fossil fuels at affordable prices [21], while the low-carbon electricity pathway faces a fundamental problem of inadequately flexible and responsive supply [22].

### Energy security evaluation

In the literature, several researchers incorporated physical availability, technology development, economic affordability, social accessibility, governance, unconventional threats, and environmental acceptability to create energy security indices [23]. A recent study analyzed the trend of 24 Asian countries from 1990 to 2014 using a comprehensive energy insecurity index based on 12 selected variables [24]. A type of assessment of energy security, using its external and internal dimensions, foreign energy supply security and the security of national energy infrastructures, was applied to trace the changing geopolitical landscape [25]. A holistic approach was proposed to evaluate energy security performance in 18 countries, including five-dimensional measures related to availability, affordability, technology development, sustainability and regulation [26]. China’s energy security was analysed from a country-wide and region-specific perspective to develop an energy system based on China’s merits [27]. An integrated order relation and entropy method was applied to evaluate China’s provincial energy security from 2010 to 2016 with three-dimensional weights (i.e., availability and stability of energy supply, sustainability and acceptability of energy use, and external influences of energy markets) [28]. A regression model with the Data Envelopment Analysis (DEA) approach was developed to evaluate energy conservation policies across 30 provinces in China [29]. A four-dimensional evaluation indexing system (i.e., resource availability, resource affordability, economic efficiency, and environmental impacts) was proposed to evaluate China’s energy security from 1953 to 2015 [30]. A so-called Chinese Energy Security Index (CESI) method was introduced to compromise three themes (i.e., energy supply dimension, economic-technical dimension, and environmental dimension) of energy security [31].

### Energy security strategies and policies

Energy strategy planning involves a persistent process of re-evaluating energy strategies. In the trade dispute, China has to seek a new energy security strategy for prevention or mitigation of incoming energy threats [32]. Energy sovereignty emphasizes the decision-making on energy systems that foster greater reliance on renewable energy to reduce multiple externalities of the fossil-fuel energy system [33]. The classical approach to energy security ensures a sufficient, uninterrupted energy supply while reducing dependence on foreign resources [34]. With the vulnerable dependence on foreign oil and gas, China’s confidence in energy self-sufficiency relies heavily on coal mining resources, because the widely distributed coal mineral deposits and railroad networks facilitate coal production and transportation [35]. The extraction of gas and oil from previously inaccessible reservoirs has been an energy game changer [36]. The shale-gas boom has made the US a globally significant producer of crude oil and natural gas with an acceptable low well-head cost [37]. Envisioning societal futures for a low-carbon systemic transition is crucial for the new configurations of the energy system [38]. China’s investment in nuclear-power generation projects has reached ¥43.7 billion in 2018 and ¥33.5 billion in 2019 [39]. The renewable power sector also showed greater resilience than fossil fuel during the COVID-19 pandemic, as renewable electricity was the only source of power generation to grow in 2020. Economies with limited fossil fuel reserves will be more affordable as more capital-intensive power generation technologies are necessary [40]. Increasingly, more industrialized economies are shifting away from fossil fuels to renewable sources (biofuels, geothermal, hydro, solar, and wind power) in their energy mixes [41]. In addition, aggregated policies such as fiscal incentives, market-based instruments, grants and research deployment are important to improve the renewable energy industry [42]. For proposing better climate policies, a stochastic decision model is developed to assess the competitive dynamics of renewable energy and fossil fuels globally [43].

### Energy security and geopolitics

The energy security game among major countries is both the confrontation of energy technology level and the cognition of national geopolitics level. Since the twentieth century, energy security has been intimately linked to geopolitics. Fossil fuel security focuses on supply disruption because of oil price volatility, the political instability of suppliers, and carbon emission reduction policies [44]. Crude oil is an indispensable strategic commodity for economic development and national defence [45]. An integrated quantitative assessment framework identifies the effects of crude oil import disruptions from a supply chain perspective, with southwestern and eastern China vulnerable to potential disruptions in oil supply. As most of China’s energy importation is through sea lanes, which are mainly under the control of the US and allies, China has diversified its importation of oil to avoid cutting off the energy supply in case of potential conflicts with the US [46]. Taking the historical lesson of World War II (e.g., the US–Japan relationship in 1941), the US would block the passage of its enemy’s energy trade. Nowadays, the US and its allies would consider to cut China’s offshore oil lines as a strategic option [47]. It is of vulnerability that China’s energy transportation line is under the control of the US Navy, resulting in the risk of cutting off oil and gas supply [48]. In particular, oil and gas are now economic and security concerns for China’s regime and key international stakeholders: (i) despite China’s increasing investments in overseas oil fields, only 10% of the import demand of oil is safe so far; (ii) the bulk of oil and natural gas supplies will mainly come from politically unstable countries and regions; and (iii) the territorial disputes with neighbouring countries regarding gas and oil reserves are ongoing [49].

Based on the above literature review, there are some studies from the perspective of energy geopolitics, energy consumption, energy import and export, energy investment, energy transportation, energy structure, low-carbon policy, etc. However, there are rare publications on energy security from the outlook of the current China-US trade dispute and the COVID-19 pandemic. To the best of our knowledge, few scholars have focused on the latest features of China’s energy security and systematically analysed how to enhance energy security to maintain economic sustainability based on real-world data. In the literature, academic research on energy security frameworks has seldom evaluated the impacts of the current crisis on China’s geopolitical and economic characteristics like foreign exchange reserves, national debt, profits from industrial enterprises, and energy trade settlement. To fill this research gap, we specifically investigate China’s energy policies and patterns based on the analysis of public data from China’s National Bureau of Statistics (http://www.stats.gov.cn/english), State Administration of Foreign Exchange (https://www.safe.gov.cn/en/), General Administration of Customs (http://english.customs.gov.cn), the People’s Bank of China (http://www.pbc.gov.cn/en/3688006/index.html), and Ministry of Finance (http://www.mof.gov.cn/en/).

## Analysis methods for China’s energy security

The PESTEL (political, economic, social, technical, environmental and legislative) model is a popular business analytical model that considers six critical business factors (i.e., political, economic, socio-cultural, technological, environmental, and legal). The PESTEL model also enables companies to monitor their operating environment in order to launch a new project. With the PESTEL model, we examine China’s geopolitical energy engagement in global energy markets, increasingly active energy diplomacy, and concrete efforts to maintain national exchange reserves. Specifically, we illustrate why China’s economy cannot afford the high price of oil and gas in the long term. The SWOT (Strength, Weaknesses, Opportunities, and Threats) analysis is another useful tool to evaluate a company’s competitive position and strategic planning. The traditional SWOT analysis has been applied in many organizations with a descriptive list of strengths, weaknesses, opportunities, and threats [50]. In this study, the PESTEL and SWOT are integrated to diagnose critical factors of China’s current and future energy security status.

### PESTEL and SWOT analysis

#### Political factors

*Foreign policy (Threat)* China’s foreign policy has been rigid at the expense of geopolitical interests. China has abandoned the isolated foreign policy to receive a larger share of economic and diplomatic engagements worldwide. In contrast, a series of changes in the “America First” strategy have not received due attention from China. An illusion that the American capitalist elite will halt the trade dispute for the sake of negotiating tempting tokens after US president election is still remaining. However, we should not ignore the possibility of bipartisan unity in the US and a cold-war reaction to an emerging opponent. In the context of the trade dispute, injurious foreign relation between China and US will be a serious threat to Chin’s energy security.

*Geopolitical relations (threat)* Volatile geopolitics have made China’s energy supply system more vulnerable. In Asia, China and US provoked competition between the US “Trans-Pacific Partnership” (TPP) strategy and China’s “One-Belt One-Road” initiative [51]. In 2019, the US Department of Defense officially released the Indo-Pacific strategy report and thus Japan recently created a special department for Indo-Pacific strategic affairs following in the footsteps of US. Because of India-China border clashes, India regards military deterrence and tactical harassment in southern Tibet as a stepping stone to joining the American military and economy. Central Asia is China’s important oil and gas exporter, through China-Kazakhstan oil and gas pipelines. In 2019, the Central Asian Gas Pipeline delivered over 47.9 billion cubic meters of natural gas to China, mainly from Turkmenistan, and in part from Uzbekistan, Kazakhstan, etc. The overland energy network, supplied primarily by these Central Asian countries, is part of the Shanghai Cooperation Organization (SCO) and the “One-Belt One-Road” initiative [52]. In the Middle East, the geopolitical landscape is also in a fragile balance. Whichever pathway the energy system follows, the world still relies on oil supply from the Middle East [4]. The Gulf countries possess 65% of the world’s crude oil reserves. In contrast, China lacks adequate reserves of crude oil and natural gas to satisfy its energy demand and sustain its economic development. Taking a geopolitical approach, China pursues its interests in trade, investment, and energy, while the United States protects its allies and tries to maintain regional leadership in the Middle East [53]. Because of the atheist ideology, China cannot establish reliable strategic alliances with Middle East countries. In Africa, Angola is China’s second-largest oil exporter. China has put its hands in the provision of infrastructure support for Angolan oil production by using oil resources as international debt repayment. In South America, Venezuela has the largest proven oil reserves in the world and has great potential as a supplement to Middle East oil. However, the extremely precarious social and political situation in Venezuela affects the stability of its oil production and exports. China’s voracious appetite for Brazilian oil, iron ore, and agricultural exports is booming. In Oceania, Australia has long been China’s largest coal supplier. However, Australia has imposed tariffs on many commodities and raised the price of coal and iron ore to China to keep pace with the US. In Europe, Russia is an energy superpower with a robust political agenda that uses energy weapons to engage in policy with China and other energy-hungry countries like Japan and Germany. Russia’s resurgence depends on energy resources, with an aspiration to become an energy superpower to augment its global influence [54]. Russia uses energy resources and pipeline monopolies as a political instrument to enforce its economic and geopolitical interests [55]. Russia will play a geopolitical role in maximizing its interests when China confronts the US. As the US continues to impose investment restrictions on China, the EU occupies a prominent place with the rapid growth of the outward foreign direct investment (OFDI) scheme in China [56].

*Trade policy (threat)* In retrospect, after imposing sanctions on ZTE and Huawei, the US imposed sanctions on entire chips and semiconductors, before joining the EU ban on China’s high-end chip manufacturing technology and equipment. Gradually, virtually all major state-owned enterprises and related companies in China are subject to sanctions listed behind closed doors. The decoupling of chips, semiconductors, and precision machine tools from the supply chain is already on the table.

*Stability of political system (strength)* In comparison, China bears a stabler government that will keep on track with the energy turnaround. China’s stable political conditions may take the initiative to ease tensions and overcome difficulties in the energy transition process.

*Fiscal and financial incentives (strength)* It has been a long-standing practice in China to promote renewable energy. High initial investment costs of hydro, nuclear, solar and wind power plants require fiscal incentives through grants, tax credits, and rebates. For instance, China provides subsidies for using on-site renewables to reduce energy poverty and provide fair access to energy services.

*Governmental regulation (strength)* If China’s government is likely to make all-encompassing reforms on regulations for renewable energy, carbon tax, and tariff of renewable energy equipment and technology. For example, Shanghai has implemented the so-called low-emission vehicle zones (LEZs) where the EV charger mandates for new buildings have become more commonplace. Since 2020, the annual report of the Chinese central government has announced the BAAS (battery as a service) model and decisively supported the infrastructure construction of switch-battery stations.

*Level of bureaucracy (weakness)* Bureaucracy in China would make it difficult to scale up renewable energy in some places. The legal and regulatory framework for producing, transmitting and distributing renewable energy still lacks clarity. Construction permits for solar and wind farms take a long time to be obtained due to complex approval processes. There would be a monopoly in grid connectivity equipment supply with increased costs and risks but without standards or specifications.

#### Economic factors

*Economic growth recovers (strength)* Before the COVID-19 pandemic, China was a prime role model of emerging economies, both overall and in economic growth. Particularly, China is the largest industrial power user in the entire industrial category of the planet. After the COVID-19 pandemic, the global recession is one of the biggest challenges facing the G20 economies. Because of China’s economic recovery in the first quarter of 2021, domestic power generation has remained at a high level.

*Disposable income of consumers (weakness)* Disposable income in urban areas gets close links to energy consumption and investment. As the global economy has gravely struggled in the last two years because of the COVID-19 pandemic, consumer incomes did not rise substantially in a recession. The business also needs to save money and spend less, particularly on unnecessary energy products. Many vacant houses will eliminate new subdivisions and will reduce the demand for associated construction materials like rebar, cement, glass, aluminium, and copper. These chain effects will lead to a significant decline of energy and commodity demand in the coming years.

*Mass unemployment (weakness)* Because of the trade dispute and COVID-19 pandemic, the whole world will experience a great depression and mass unemployment. To face the potential crisis, China should turn this challenge into an opportunity by pre-transforming its energy policies and patterns.

*Global energy transition (opportunity)* The global energy transition to a new energy order unfolds the gradual shifts toward low-carbon energy sources. Moreover, achieving the goal of inclusive net-zero emissions will require an unprecedented energy transition. If China continuously increases the renewable mix to 90%, it would invest up to $15 trillion for the massive shift in the next 30 years. Above all, China’s power sector is the largest contributor to the total economic investment. The emergence of enormous benefits will also speed up the establishment of a financial system for green energy. China has accelerated its considerable investment in hydroelectric (e.g., “Three Gorges Dam” project), solar, wind and nuclear power plants, making the renewable energy sector grow faster than fossil fuel plants. In addition, China’s tremendous domestic market provides a sufficient motive for the transition of energy.

*Renewable-energy cost reduction (opportunity)* Renewable energy business has obtained the benefits from decreasing the battery costs. For instance, wind and solar power have become increasingly cost-competitive in comparison to fossil fuel power plants. Additionally, the development of manufacturing facilities for solar and wind energy grows rapidly. In this situation. Prices of photovoltaic cells across the globe have declined significantly because of the increased availability of silicon.

*Battery technology advancement (opportunity)* Since advanced batteries have left the specialized markets and have entered mainstream markets, they represent a tipping point for technologies such as electric vehicles and rooftop solar boosters. Significantly, battery and micro-processing innovations have reduced the costs of other electric vehicle components. Because of the decline of battery costs, new business models for batteries emerge will provide new functions from ancillary services to on-demand services.

*Manufacturing industry outflows (threat)* China competes with some developing countries (e.g., Mexico, India, Vietnam, Indonesia, Turkey and Brazil) as rivals in low-end manufacturing. China’s industrial overcapacity depends heavily on exports to the international market. Consequently, China relies increasingly on outside economic growth. US tariff sanctions and the tightening of customs clearance will significantly increase the risk of production halts and destruction of foreign trade relationships, or even lead to disruption of the overall supply chain. It has forced China’s labour-intensive export enterprises to transfer to some resource-rich neighbouring countries (e.g., India, Vietnam, and Burma) without tariff barriers. In addition, the limited population of young workers darkens the future of Chinese manufacturing. Moreover, the COVID-19 pandemic may suffer the world for a long time, affecting the global industrial chain. Fortunately, China’s social organization and mobilization capabilities give it a temporary competitive advantage in fighting the COVID-19 outbreak to minimize the impact on the manufacturing sector.

As shown in Table 1, China is encouraging companies to resume production and increase investment in industrial fixed through a significant increase in fiscal stimulus and the release of low-cost loans. The resulting effect is also remarkable, as China’s trade surplus clearly stands at $298.9 billion in 2020. As shown in Table 2, although the Return on Equity (ROE) of industrial enterprises continues to decrease to 11.6% in 2020, the total profit of industrial enterprises increases by 4.1%. Furthermore, the newly added industrial net asset ratio reached its highest level up to 61.3% since 2015. However, the COVID-19 pandemic will force more corporations to develop onshore supply chain strategies to handle supply chain disruptions. Obviously, supply chain transfer will go hand in hand with the energy demand transfer. As an emerging manufacturing rival with a considerable population and a territory similar to China, India’s energy supply and consumption will soon reach the same magnitude as that of China.

**Table 1:** China’s fiscal status in 2008–2020

Year	Changes in total fiscal deficit				Net foreign exchange reserves			Contrast of Outstanding foreign exchange funds to the total assets of the central bank			Total trade surplus data				Net foreign investment data			
	Total fiscal revenue (¥100) million)	Total fiscal expenditure (¥100 million)	Total fiscal deficit (¥100 million)	Growth rate (%)	Net foreign exchange reserves ($100 million)	Growth rate (%)	Exchange rate (RMB/USD)	Outstanding foreign exchange funds (¥100 million)	Total assets of the central bank (¥100 million)	Ratio of funds outstanding for foreign exchange to total assets of the central bank (%)	Trade deficit in services ($100 million)	Surplus in commodity trade ($100 million)	Total trade surplus ($100 million)	Growth rate (%)	Foreign capital utilization ($100 million)	Outbound investment ($100 million)	Net foreign investment ($100 million)	Growth rate (%)
2008	61,330	62,592	1262	− 18.10	15,558	36.6	6.95	149,624	207,096	72.2	− 115	2981	2866	11.80	924	559	365	− 24.43
2009	68,518	76,299	7781	516.6	19,705	26.7	6.83	175,154	227,535	77.0	− 295	1957	1662	− 42.0	901	565	336	− 7.9
2010	119,887	123,825	3938	− 49.4	22,984	16.6	6.77	206,767	259,275	79.7	− 220	1815	1595	− 4.0	1057	688	369	9.8
2011	145,237	149,195	3958	0.5	24,861	8.2	6.46	232,389	280,978	82.7	− 549	1549	1000	− 37.3	1160	600	560	51.8
2012	154,789	143,519	− 11,270	− 384.7	25,746	3.6	6.29	236,670	294,537	80.4	− 896	2303	1407	40.7	1117	772	345	− 38.4
2013	181,478	190,713	9235	− 181.9	29,581	14.9	6.1	264,270	317,279	83.3	− 1184	2591	1407	0	1176	902	274	− 20.6
2014	194,484	203,250	8766	− 5.1	29,475	− 0.4	6.12	270,681	338,249	80.0	− 1599	3831	2232	58.6	1196	1029	167	− 39.1
2015	194,599	218,225	23,626	169.5	25,709	− 12.8	6.49	248,538	317,837	78.2	− 2065	5939	3874	73.6	1263	1180	83	− 50.3
2016	206,224	234,693	28,469	20.5	20,776	− 19.2	6.94	219,425	343,712	63.8	− 2455	5099	2644	− 31.8	1260	1701	− 441	− 631.3
2017	234,209	264,030	29,821	4.7	20,172	− 2.9	6.53	214,788	362,932	59.2	− 2555	4225	1670	− 36.8	1329	1201	128	− 129.0
2018	258,757	301,468	42,711	43.2	17,469	− 13.4	6.86	212,557	372,492	57.1	− 2915	3518	603	− 63.9	1350	1298	52	− 59.4
2019	274,898	330,239	55,341	29.6	10,506	− 39.9	7.03	212,317	371,130	57.2	− 2614	4628	2014	234.0	1567	976	591	1036.5
2020	276,384	363,587	87,203	57.6	8157	− 22.4	6.52	211,308	387,677	54.5	− 1453	5338	2989	48.4	1444	1329	114	− 80.7

(1) Data of total fiscal deficit is from China’s Ministry of Finance. (2) Data of Outstanding foreign exchange funds and the total assets of the central bank is from the People’s Bank of China. (3) Data of international trade in goods and services in terms of China’s balance of payments are from China’s State Administration of Foreign Exchange

**Table 2:** China’s industrial profits and net assets in 2012–2020

Year	Industrial profits		Industrial net assets			
	Total profits ($100 million)	Growth rate of the total profits (%)	Total net assets at the end of the period (¥100 million)	Growth rate of the total net assets (%)	The rate of return on industrial net assets (%)	Ratio of profit changing into newly-added industrial net assets (%)
2012	61,910	0.8	323,049	14.1	19.2	–
2013	68,379	10.4	365,057	13.0	18.7	61.4
2014	68,155	− 0.3	409,746	12.2	16.6	65.6
2015	66,187	− 2.9	444,088	8.4	14.9	51.9
2016	71,921	8.7	479,224	7.9	15.0	48.9
2017	74,916	4.2	493,893	3.1	15.2	19.6
2018	71,609	− 4.4	511,977	3.7	14.0	25.3
2019	61,996	− 13.4	517,426	1.1	12.0	8.8
2020	64,516	4.1	556,968	7.6	11.6	61.3

**Fig. 1:**
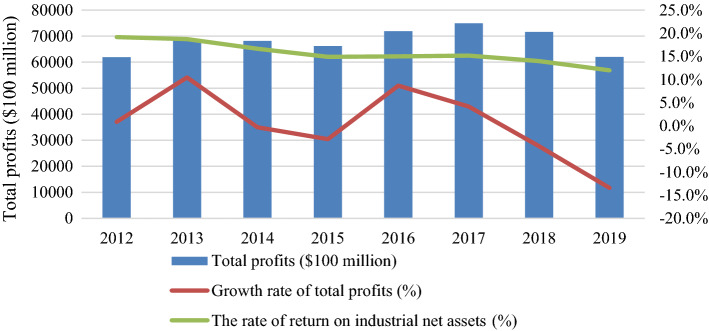
Analysis of China’s industrial profits from 2012 to 2019

**Fig. 2:**
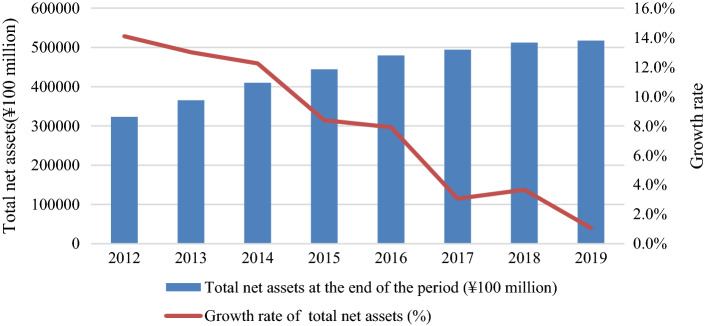
Analysis of China’s industrial net assets from 2012 to 2019

China would no longer provide cheap goods after this trade dispute and undercut its industrial competitiveness, based on the data analysis of Figs. 1, 2. Losing the “World Factory” will directly reduce China’s energy requirements for factories and logistics. In additionon, the trade conflict will also hinder China's high-tech sectors (e.g., Huawei’s 5G project). As analysed in Fig. 1, the profits of Chinese industrial companies have steadily declined over 4 years since 2016. As analysed in Fig. 2, the growth rate of net assets of industrial enterprises has virtually come to a standstill after this trade dispute, and the return rate of industrial net assets has dropped from 19.2% in 2012 to 12% in 2019. As observed in Tables 3, 4, there has been a significant impact on both China’s industrial input and output since 2018 because of the trade dispute. Many multinational companies have diversified their supply chains to find tariff-friendly sources outside China, launched or announced new manufacturing plants in the US. If the trade dispute is continuing, China’s industrial enterprises cannot afford the high price of imported fossil fuels any more.

**Table 3:** China’s primary energy imports in 2010–2019

Year	Natural gas import				Crude oil import			Refined oil import			Coal import				Total energy import cost	
	External dependence (%)	Import volume (100 million cubic meters)	Growth rate (%)	Import cost ($100 million)	External dependence (%)	Import quantity (10 thousand tons)	Growth rate (%)	Import cost ($100 million)	Import quantity (10 thousand tons)	Import amount ($100 million)	External dependence (%)	Import quantity (million tons)	Growth rate (%)	Import cost ($100 million)	Total cost for energy imports ($100 million)	Growth rate (%)
2010	11.7	165	–	–	54.8	23768	–	1353.1	3688	224.7	4.7	1.63	–	169	–	–
2011	21.6	312	89.1	–	56.5	25378	6.8	1966.6	4060	327.8	5.8	2.22	36.2	209.1	–	–
2012	27.1	421	34.9	161.8	56.1	27103	6.8	2206.6	3982	329.9	7.5	2.88	29.7	287.1	2985.4	–
2013	27.3	525	24.7	106.3	58.1	28194	4.0	2196.5	3959	319.3	8.3	3.27	13.5	290	2912.1	− 2.5
2014	26.4	591	12.6	122.2	59.6	30837	9.4	2283.1	2999	234.3	7.5	2.92	− 10.7	222.5	2862.1	− 1.7
2015	32.1	611	3.4	88.1	60.5	33550	8.8	1344.5	2990	143	5.3	2.04	− 30.1	121	1696.6	− 40.7
2016	35.9	721	18.0	164.9	64.4	38101	13.6	1164.7	2784	111.4	6.8	2.56	25.5	141.5	1582.5	− 6.7
2017	39.1	920	27.6	232.8	67.4	41957	10.1	1623.3	2964	144.8	7.1	2.71	5.9	226.4	2227.3	40.7
2018	45.3	1254	36.3	461.9	70.9	46190	10.1	2402.6	3348	200.7	7.4	2.82	4.1	246.1	3311.3	48.7
2019	43.4	1340	6.9	417.2	72.0	50572	9.5	2389.7	3056	170.8	10.7	3.00	6.3	220.3	3197.9	− 3.4

**Table 4:** China’s primary energy consumption in 2010–2020

Year	Primary energy consumption (10 thousand tons standard coal)					Proportion of primary energy consumption (%)				Primary energy production (10 thousand tons standard coal)					Proportion of primary energy production (%)			
	The total energy	Coal	Oil	Natural gas	Hydro power, nuclear power, wind power, etc	Coal	Oil	Natural gas	Hydro power, nuclear power, wind power, etc	The total energy	Coal	Oil	Natural gas	Hydro power, nuclear power, wind power, etc	Coal	Oil	Natural gas	Hydro power, nuclear power, wind power, etc
2010	360,648	249,568	62,753	14,426	33,901	69.2	17.4	4.0	9.4	312,125	237,839	29,028	12,797	32,461	76.2	9.3	4.1	10.4
2011	387,043	271,704	65,023	17,804	32,512	70.2	16.8	4.6	8.4	340,178	264,658	29,838	13,947	32,657	77.8	8.5	4.1	9.6
2012	402,183	275,465	68,363	19,303	39,007	68.5	17	4.8	9.7	351,041	267,493	28,915	14,393	39,317	76.2	8.5	4.1	11.2
2013	416,913	280,999	71,292	22,096	42,525	67.4	17.1	5.3	10.2	358,784	270,523	30,138	15,786	42,336	75.4	8.4	4.4	11.8
2014	425,806	279,329	74,090	24,271	48,116	65.6	17.4	5.7	11.3	362,212	266,333	30,397	17,008	48,128	73.6	8.4	4.7	13.5
2015	429,905	273,849	78,672	25,364	52,019	63.7	18.3	5.9	12.1	362,193	260,986	30,725	17,351	52,414	72.2	8.5	4.8	14.5
2016	435,819	270,320	79,788	27,904	57,988	62.0	18.3	6.4	13.3	345,954	240,816	28,372	18,338	58,474	69.6	8.2	5.2	16.7
2017	448,529	270,912	84,323	31,397	61,897	60.4	18.8	7.2	13.6	358,867	246,434	27,143	19,607	65,817	68.6	7.6	5.4	17.4
2018	464,000	273,760	87,696	36,192	66,352	59.0	18.9	8.0	14.1	378,859	257,522	26,979	21,316	71,183	68.3	7.2	5.4	18.2
2019	487,488	281,281	92,623	38,999	74,586	57.7	19.0	8.0	15.3	397,317	272,162	27,415	22,250	75,490	68.5	6.9	5.6	19.0
2020	498,000	282,864	94,122	41,832	79,182	56.8	18.9	8.4	15.9	408,000	275,808	27,744	24,480	79,968	67.6	6.8	6.0	19.6

*Inflation rates and property bubble (Threat)* Over the last 30 years, a thriving local real estate industry has anchored China’s government revenues. The Chinese central bank kept interest rates low to stimulate economic growth during the recession. However, these low-interest loans have sunk into housing securities, particularly for mortgages. In particular, it has already amplified the wealth gaps, raised labor costs, crushed domestic demands, and exposed investments in risky loans.

**Fig. 3:**
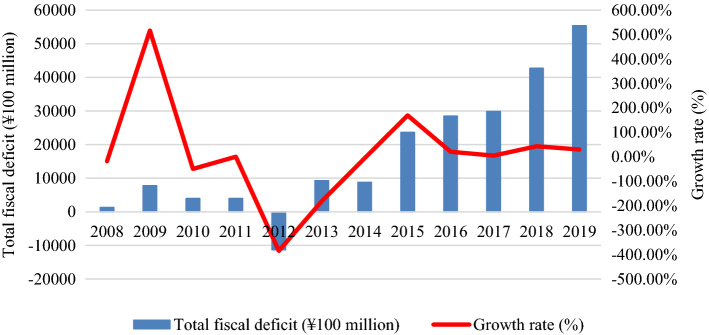
Analysis of China’s fiscal deficits from 2008 to 2019

**Fig. 4:**
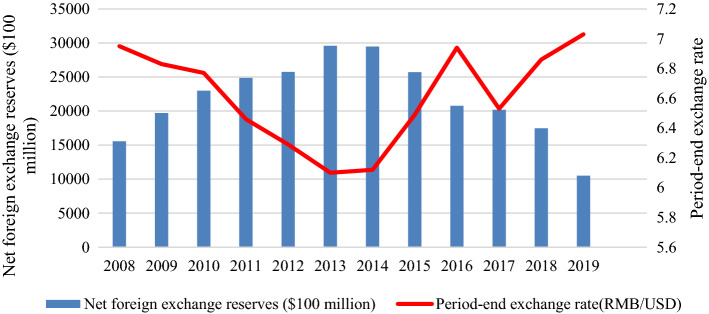
Analysis of China’s net foreign exchange reserves from 2008 to 2019

*Government budget deficits and foreign exchange reserves (Weakness)* China is facing challenges in government revenues, foreign exchange reserves, and budget allocation when it seeks to invest in renewable energy. As shown in Table 3, China spent over $300 billion on oil, gas, and coal purchases annually from 2018. According to the analysis in Fig. 3, China must maintain an annual trade surplus of over $650 billion, because it requires about $300 billion for energy imports, over $300 billion for chips, and over $50 billion for agricultural products annually. Otherwise, China will confront the dilemma of constantly depleting foreign exchange reserves. Because of the decline of both industrial profits and net foreign exchange reserves, as analysed in Fig. 4, China will not bear high energy prices and more oil and gas imports. China’s net foreign investment has decreased, because China has launched various infrastructure projects to initiate the “One-Belt, One-Road” since 2013. In the Q1 of 2021, China’s external debt outstanding amounted to $2,526.6 billion, while the foreign reserve balance was $3,170 billion. As a result, China’s net reserves fell to $643.4 billion. Table 3 shows China’s heavy dependence on foreign exchange reserves for energy security, which is indirectly linked to international trade and foreign capital inflow. If the RMB continues to be appreciated, China’s export earnings will suffer significantly. The value of the RMB will progressively lose its support through foreign exchange reserves and face a risk of depreciation. The weaker RMB would encourage exports but will raise domestic fossil energy prices because of imported inflation and more trade friction with other countries. The trade dispute will reduce the export market for manufactured goods from China and lead to the overcapacity of the Chinese manufacturing industry. As indicated in Table 4, the rapid growth in energy consumption has led to enormous budget expenditures and extravagant infrastructure in China.

*Markets of alternative energy vehicles (Strength)* China is providing considerable support for the Alternative Energy Vehicles (AEVs), which use electricity by rechargeable batteries, fuel cells, hydrogen, or hybrids. Electric Vehicle Manufacturers such as Tesla timely entered China’s market, which was least affected by the global recession caused by the COVID-19 pandemic. The huge market of AVEs enables China to join the high prosperity of the electric vehicle industry and intelligent traffic systems. In 2020, global sales of electric and hybrid vehicles will reach 3.24 million units, achieving a 43% increase over 2019. Noticeably, China has become the largest renewable vehicle market, accounting for 41% of global sales. Electric car sales continued to soar in 2020, with a global growth of 38% and a domestic growth of 13.3% in China. In essence, electro mobility represents a future energy pattern different from fossil fuel consumption.

*Shortage of battery metals (Threat)* COVID-19 pandemic halted production of most metals last year, while investments in renewables and electric vehicles will continue to grow in 2021. Specifically, there are over 70% of the world’s electrical lithium-ion batteries made in China. In the first quarter of 2021, China’s battery enterprises invested in about 20 new battery-related projects, totalling over ¥160 billion. Besides, it drives a surge in global demand for battery metals. Thus, the prices of copper, cobalt, lithium, tin and other battery metals have surged. China should accrue ore materials like cobalt and lithium as strategic reserves.

#### Social Factors

*Attitudes towards carbon emissions (Opportunity)* Public support for renewables could speed up the transition. Fossil fuels contribute to pollution by emitting CO2 and other greenhouse gases. While US energy-related CO2 emissions continue to decrease, the Chinese government has encouraged low-carbon lifestyles. Therefore, to reduce their environmental impact, customers are increasingly concerned about the effects of automotive emissions on air quality.

#### Technological factors

*Access to new energy technologies (Opportunity)* Advancements in energy science and technology are multifaceted. New energy technologies, such as electric vehicles and automatic drive, result from technological innovation, which will advance and accommodate the standards of performance accepted by the customers. In China, these new energy technologies are bringing an opportunity for industrial upgrade.

*Trend of electrifying transportation (opportunity)* At present, petrol and diesel vehicles are more economical for customers than electric ones. By the end of 2020, the number of electric vehicles in China was 4.92 million, with a total 23GWh of retired power batteries. Furthermore, a future alternative to almost all public vehicles may be electric transportation.

*R&D on energy technologies (Weakness)* China’s weak innovative processes resulted from a lack of innovation and creativity in education. In fact, with the accretion of renewables, supply chain security of renewable energy facilities has taken on a new connotation. For example, a high cost of imports (e.g., wind power converter, main rotor bearing, gearbox bearing) contributes to the high price of wind power equipment in China. This situation will decrease the self-sufficiency of the renewable-energy economy. Thus, China should invest more R&D funds to accomplish breakthroughs in key energy technologies.

*Smart power technology incentives (Opportunity)* Electricity is moving to the heart of modern energy security because of the cost reductions of renewable energy. In addition, the reduction of carbon emissions requires harnessing the potential of the digital energy economy. Advances in digital technologies are opening up amazing opportunities for energy transition. The data on energy development, production, and appliances are shared in a “cloud” base to achieve the aggregation and affiliation of energy flow, information flow and capital flow, resulting in enhancing the predictability and accuracy of energy security.

#### Environmental factors

*Environmental pollution (Threat)* In many cities of China, emissions from factories and automobiles frequently cause toxic smog that lasts for days or weeks. Smog has blanketed more than a quarter of China’s territory, leaving most cities with a hazy sky. In addition, other pollutions such as oil and waste flows into the seas are poisonous to marine organism. In this case, China must take more initiatives to reduce carbon emission and protect environment for future generations.

*Climate change and sustainability (Threat)* Because of high variability in energy supply, sustainable development is the cornerstone of energy security issues. China’s official media and press need to promote social education and increase public awareness on environmental sustainability.

#### Legal Factors

*National and municipal commitments (Strength)* The government and its policy commitments play an essential role in the development of renewable energy. City-level renewable energy policies have expanded beyond the national level. To achieve carbon neutrality, China has drafted the 14th Five-Year Plan, which will have a profound impact on the future energy transition based on these national and municipal commitments.

*Lack of renewable energy patents (Weakness)* Patent, intellectual property and trademark protection are becoming more important in China but also cause more legal issues. Globally, US companies still hold the most patents on renewable energy technologies. Through various licensing agreements with foreign counterparts, Chinese energy companies would be facing their patent disadvantages.

In conclusion, a SWOT analysis of China’s energy security factors is summarised in Table 5.

**Table 5:** A SWOT analysis of China’s energy security factors

	Support	Oppose
Internal	Strength	Weakness
	Stability of political system	Level of bureaucracy
	Fiscal and financial incentives	Disposable income of consumers
	Governmental regulation	Mass unemployment
	Economic growth recovers	Government budget deficits and foreign exchange reserves
	Markets of alternative energy vehicles	R&D on energy technologies
	National and municipal commitments	Lack of renewable energy patents
External	Opportunity	Threat
	Global energy economic transition	Foreign policy
	Renewable-energy cost reduction	Geopolitical relations
	Battery technology advancement	Trade policy
	Attitudes towards carbon emissions	Shortage of battery metals
	Energy technological change	Inflation rates and property bubble
	Access to new energy technology	Manufacturing industry outflows
	Trend of electrifying transportation	Environmental pollution
	Smart power technology incentives	Climate changes and sustainability

### Game analysis and QSPM

This section aims to determine the preferable energy security strategy during the trade dispute, by conducting a bipartite game reciprocity model and a QSPM (Quantitative Strategic Planning Matrix) analysis. This game analysis model helps us analyze the consequent evolution of contradictions from China’s national energy structure, achieving a coherent comparison of strategic interactions between China and the US. Apparently, the avoidance of strictly dominated strategies of remaining a present energy pattern, which is a minimum requirement of economic rationality. The best energy security strategy for China is to maintain strategic cooperation with the US. However, adopting a cooperative strategy with the US would cause China’s other security outcomes. In fact, the so-called “US fear” about its declining hegemony and China’s rapid rise as a big challenger is driving a US-launched trade dispute with China. Indeed, the underlying cause of the trade dispute is political rather than economic. Cooperation with the US rather than confrontation has always been China’s best option to maintain economic development and energy security.

The QSPM is a strategic management tool used in the evaluation of strategic options and determination of relative attractiveness of strategies. The QSPM provides an analytical method for comparing workable alternative actions and prioritizes these strategies. In this study, we apply the QSPM to assess China’s energy security polices and strategic options. As shown in Table 6, rather than conflicting with each other, cooperation between these two countries has inherent advantages in line with their energy security interests. Our results also reaffirm the importance of transition into renewable energy. We propose a qualitative framework for analysing the energy transition in the Chinese government agenda. In order to conduct this study, we collected data from the State Statistics Service of China. Using the QSPM matrix analysis, we found it from Table 7 that China should adopt the strategy of transitioning to renewable energy and keeping nuclear power as a part of its energy mix. Using renewable electricity at large scale will reduce energy intensity, replacing bulk coal, liquefied petroleum gas, etc. Battery technology is mature enough to make electricity cheaply stored for a long period. The transformation of the energy consumption structure of electric energy is also conducive to the development of information, automation and intelligence of the economy. The benefits of renewable energy include low carbon and low pollution, which will also help reduce the overall cost of energy. In addition, it can reduce the consumption of China’s foreign exchange reserves and provide neighbouring countries with a certain amount of electricity to earn foreign exchange. However, China’s development still relies on government financial support and requires long-term government investment. China’s stable political state, strong national control and non-market fiscal subsidies.

**Table 6:** Game analysis of energy security under the condition of China–US trade dispute or cooperation

Provincial energy security index system	Transform into renewables and nuclear energy				Remain the present energy pattern			
	The USA out of trade-war	The USA in trade-war	China out of trade-war	China in trade-war	The USA out of trade-war	The USA in trade-war	China out of trade-war	China in trade-war
Availability and stability of energy supply structure (positive indicator)								
Production and consumption Ratio (PCR)	1	0	1	1	1	0	0	− 1
Production Diversity Index (PDI)	1	− 1	1	1	− 1	− 1	0	− 1
Storage and production ratio	1	1	0	− 1	1	1	0	− 1
Renewable energy ratio	1	− 1	1	1	− 1	− 1	0	1
Energy transportation	1	0	1	0	1	0	1	− 1
Sustainability and acceptability of energy use (negative indicator)								
Energy intensity	1	0	1	1	1	− 1	0	− 1
The structure of terminal energy consumption	1	0	1	1	1	− 1	1	− 1
Carbon intensity per unit of energy	1	0	1	1	1	− 1	1	− 1
Sewage ratio	1	0	1	1	1	− 1	1	− 1
External influences of policies and markets (positive indicator)								
Energy price volatility improve	1	0	1	− 1	1	0	1	− 1
The nation with more dollar reserve to trade energy	1	− 1	1	− 1	1	− 1	1	− 1
The enterprises with more profits to afford energy payment	1	− 1	1	− 1	1	− 1	1	− 1
Energy policy with more financial support	1	− 1	1	1	1	− 1	1	− 1
More investment in the energy industry	1	1	1	1	0	0	0	0
Sum	14	− 3	12	5	9	− 8	4	− 11

**Table 7:** A quantitative strategic planning matrix (QSPM) analysis for China’s energy security strategy

Key factors	Weighs	Transform into renewable energy				Transform into oil and gas				Transform into nuclear power			
		China out of trade dispute		China in trade dispute		China out of trade dispute		China in trade dispute		China out of trade dispute		China in trade dispute	
		AS	TAS	AS	TAS	AS	TAS	AS	TAS	AS	TAS	AS	TAS
Strengths (IF)													
The level of national energy independence by the production and consumption ratio	0.14	4	0.56	3	0.42	2	0.28	1	0.14	4	0.56	3	0.42
Energy policy with more financial and fiscal support	0.04	3	0.12	2	0.08	3	0.12	1	0.04	3	0.12	2	0.08
Weaknesses (IF)													
Low level of energy diversification by the Production Diversity Index (PDI)	0.12	4	0.48	4	0.48	2	0.24	1	0.12	2	0.24	2	0.24
Limited energy reserves by the storage and production ratio	0.20	2	0.40	1	0.20	2	0.40	1	0.20	2	0.40	1	0.20
High level of pollution by the sewage ratio	0.04	3	0.12	3	0.12	2	0.08	1	0.04	2	0.08	2	0.08
Weakly innovative processes in energy efficiency by the energy intensity	0.09	3	0.27	3	0.27	2	0.18	1	0.09	3	0.27	3	0.27
High carbon intensity per unit of energy	0.04	3	0.12	3	0.12	2	0.08	1	0.04	3	0.12	3	0.12
Equitable access to energy services by the structure of terminal energy consumption	0.05	3	0.15	3	0.15	3	0.15	1	0.05	3	0.15	3	0.15
Threats (EF)													
The geopolitical relations affecting energy transportation	0.09	3	0.27	3	0.27	3	0.27	1	0.09	3	0.27	2	0.18
Vulnerably secure supply of oil and gas caused energy price volatility	0.03	3	0.09	1	0.03	3	0.09	4	0.12	4	0.12	2	0.06
The nation getting enough dollar reserve to trade energy	0.04	3	0.12	1	0.04	3	0.12	1	0.04	3	0.12	1	0.04
Opportunities (EF)													
More investment in the energy industry	0.04	4	0.16	2	0.08	3	0.12	2	0.08	3	0.12	2	0.08
Reduction of renewables cost and battery cost stimulating renewable energy ratio	0.08	4	0.32	4	0.32	2	0.16	3	0.24	1	0.08	3	0.24
Total	1		3.18		2.58		2.29		1.29		2.65		2.16

Because of the combined impact of the China-US trade dispute and the COVID-19 outbreak, there is considerable uncertainty about China’s production capacity and export competitiveness. There is a risk that China’s net foreign exchange reserves may continue to shrink. As a major type of infrastructure, the construction and operation of energy facilities can contribute to economic development. However, investment in renewable energy facilities can effectively stimulate economic development and bring significant employment. In particular, investments in renewable energy facilities will enable China to take the lead in the new energy revolution and efficiently boost the economy and jobs associated with sustainable development. In China, a transition to renewable energy and nuclear power will give the country a higher level of energy security. Throughout its borders, China has excellent renewable resources such as wind, hydro, geothermal, and solar power. If China relies primarily on oil and gas, it must be more seriously dependent on the fragility of outer geography for oil and gas supply. The cost of producing and storing renewable energy is rapidly declining, and the availability of charging facilities is fueling the spread of mobile devices and electric vehicles even faster. Large-scale development and advances in renewable energy will also bring electricity prices below the cost of oil and natural gas, which will help China reduce the cost of manufacturing and enhance its national competitiveness.

## Policy and managerial implications

Based on the above analysis, we propose some policy and managerial implications for improving China’s energy security in this section.

### Diversification of energy structure

There is bidirectional causality between the following pairs: international trade and energy demand; capital flow and industrial investment, financial expansion and economic growth; international trade and business prosperity. In the same fashion, many export-oriented sectors in China are not only energy intensive but also labor intensive. Energy polices affect the energy structure through the official regulations such as energy patterns, energy investment, energy subsidy, adjustment of tariffs and currency exchange rates. In the coming years, China’s energy patterns will face a huge transformation opportunity or challenge, as the electrification level in end-user sectors will surge and the electricity energy ratio will rise substantially due to the increasing awareness of environmental protection and green development. China would tempt to improve its industrial infrastructure, develop new sources of energy supply, announce energy conservation measures, and engage in a more aggressive energy diplomacy to diversify its energy supply chain. Facing the trade dispute, a primary concern for China would be the uncertain external environment, e.g., whether trading contracts could be retained to satisfy China’s growing energy demand. Most oil purchase orders of China are concentrated in a few geographically-unfriendly countries. One of crucial links between China and its allied countries is energy trading. However, such an energy-trading pattern is actually fragile as these countries (e.g., Iran and Venezuela) are facing political and economic instability because of trade and financial sanctions from the USA. It is of great significance for China’s manufacturing industry to maintain energy-trading contracts with these oil-producing countries. One main purpose of the well-known “One-Belt and One-Road” initiative is to strengthen cooperation with energy-rich nations (e.g., Central Asia such as Kazakhstan and the Middle East countries such as Iran), mainly for establishing a more secure and more sustainable overland/oceanic energy supply chain network.

### Exploitation of other energy sources

China’s tremendous domestic market provides sufficient motives for transformation of energy patterns. The Gulf countries possess 65 per cent of the world’s crude oil reserves. In comparison, China lacks enough reserves of crude oil and natural gas to satisfy its energy demand and sustain its economic development. China’s shale gas technology is developing but is still fallen behind due to its inefficient energy patterns. Moreover, over three-fifths of shale oil and gas resources locate in water-deficient areas and seismic zones. With the vulnerable dependence on foreign oil and gas, China’s confidence of energy self-sufficiency heavily relies on coal mining resources, as the widely-distributed coal mineral deposits and accessible railway networks can facilitate production and transportation of coal. In addition to coal energy, hydro-power, solar-power and nuclear-power plants will form the backbone of China’s energy security in the future. In comparison to the hydro-power capacity that has reached a limit, there is more room for the growth of solar-power and nuclear-power plants. At present, China is one of the largest nuclear-power-user countries and contributes to around three quarters of the total global growth of nuclear-power generation. In the long-term cooperation with vendors of nuclear technology such as Areva and Westinghouse that are respectively French and American multinational companies specializing in nuclear power, Chinese engineers have mastered the safer and lower-cost technology for construction of new nuclear-power plants. At the same time, China’s state-holding enterprises have invested in uranium-rich countries such as Namibia to hold several large-scale uranium mines. However, nuclear waste disposal and nuclear leakage by accident are the potential hazards that must be seriously measured in China’s current energy pattern transformation. To meet the energy demands, more efforts should be made to transform national energy patterns.

### Increase of natural gas supply

Since 2008, China’s economy was overheated mainly owing to the so-called “4 trillion Infrastructure Investment Plan”. As a result, the total amount of domestic energy supplies was unable to meet the actual energy demand. China had to purchase more crude oil, natural gas and coal from the international energy market. One of main driving forces of international oil price comes from the fast-growing energy demand of China. As shown in Table 3, more than $280 billion US dollars was consumed for buying oil, gas and coal per year on average from 2013 to 2015. At the same time, coal mining investment continuously declined in 5 years from 2013 to 2017. In contrast, the sharp increase of natural gas demand in China pushed the rise in the supply of global natural gas. There was a shortage of natural gas even in port cities such as Shanghai due to the limitation of shipment capacity so that no excess natural gas can be shared with and transported to inland cities. The production capacity of natural gas in the western cities of China is still so low that the amount of gas transmitted to the middle and the eastern cities of China is insufficient to meet the demand. Due to the regulation of environmental protection, many factories were closed down for stopping the use of coal-fired boilers since 2015. In some cities, residents can only consume natural gas instead of coal to meet their daily energy needs such as cooking or heating. In this case, it is nearly impossible for China to ensure energy self-sufficiency and has to rely on the international energy market to fulfil energy gaps, especially for natural gas. If the capability of earning foreign exchanges through exports is abated, the imbalance between insufficient gas supply and growing energy demand would create an ongoing threat to China’s energy security, environmental protection, economic development and social welfare.

### Energy infrastructure upgrade for manufacturing industry

In the context of the trade dispute, China should enhance crisis consciousness because the possible shortage of energy imports will result in a serious energy crisis. As China’s export-oriented manufacturing industry is sensitive to the energy price, the rise in energy cost will weaken its competitiveness, reduce its export capacity and squeeze its international market share. Therefore, it is essential for China to find the feasible solution to guarantee security and sustainability of energy supply. China should maintain reasonable diplomatic relations with major energy suppliers. China makes the competition with some rival developing countries (e.g., Mexico, India, Vietnam, Indonesia and Brazil) in the low-end manufacturing industry. Insufficient energy supply will increase the manufacturing cost and then lead to disruption of the overall supply chain. The expansion of the manufacturing industry is always followed by the inflow of capital, employment and urbanization in China and these rival developing countries, which will boost the energy needs rapidly. Due to tariff sanctions by the USA, a large number of labor-intensive manufacturing enterprises have already moved or are moving to burgeoning resource-rich Asian countries such as India, Vietnam, Indonesia and Burma. India has a larger population of young labors in comparison to China and is accelerating the improvement of industrial infrastructure in order to replace China as the next world factory. If the trade dispute is continuing, China would undercut its industrial competitiveness and lose the title of “World Factory” presumably. To face these challenges, the national energy infrastructure such as the coal/gas supply chain networks, smart high-voltage power grid system and public charging facilities should be upgraded and supported by more national energy funds.

### Advancement of electrical power system

Due to the high variability in supplies, sustainability is the cornerstone of future energy security issues. It is estimated that the energy source of almost all public transportation vehicles will come from electrical power. A digital internet-of-things (IOTs) economy will connect nearly all consumers’ devices and appliances. To achieve this aim for China, it will require more reforms to improve the overall energy patterns, more investments to upgrade national electricity grid systems, more promotion to advance battery storage and charging technologies. In China, the power sector accounts for the most important proportion of the total energy investment. The power network is foreseeable to serve the digital economy and the smart economy in the near future, especially the ultra-fast storage and instant charging network of various smart devices. However, the switch from the classical petroleum energy system to the new electricity energy system still needs enormous investment, practically for advancing technological innovations (e.g., electric-power vehicles, autonomous trucks, driverless metros, fabrication robots, next-generation mobile communication systems) as well as management progression (e.g., big-data-driven AI and optimization software).

### Integration with global energy market

In the trade dispute, the restriction of energy imports would result in the decline of energy demand for China. The increase in tariffs and the stricter customs clearance significantly increase the risk of production halts and foreign trade contract breaches. It has forced a large number of factories to move from China to non-tariff or low-tariff countries such as Vietnam. As a result, China’s manufacturing industry will lose its horsepower and slide into the contraction of energy demands, industrial investment and employment. As indicated in Tables 3, 4, the statistical data show that there is a significant impact of the trade dispute on both China’s industrial input and output since 2018. The transfer of the supply chain will be accompanied by the transfer of energy demand. Because of the trade dispute, China’s labor-intensive export enterprises have been transferred to some neighboring countries (e.g., India, Vietnam and Burma) without tariff barriers. At the same time, the demand for transportation tools will be decreased, resulting in a sharp decline of sales in the automotive industry which is the largest industrial sector in China. The income of residents is closely related to energy consumption, and the energy demand of industrial population is greater than that of agricultural population. Consequently, the climbing of China’s unemployment rate caused will further lead to a decline in resident income and energy demand. The unemployed workers, who have registered in rural areas with no adequate social security in the cities, will have to return to the countryside with less energy demand. These chain-effect factors will lead to a significant decline of energy demand in the coming years. To face the potential crisis, Chinese energy companies should actively penetrate the global energy market and become a major member of the energy supply chain, e.g., procuring some reliable offshore energy companies with indirect holdings or multi-layer holdings.

### Establishment of a new energy trading platform

The trading platform for renewable energy suppliers represents a great opportunity for China. Currently, China’s international energy trade depends on the US dollar as a major settlement currency. China has an unavoidable dollar reserve to guarantee the security of energy trade. Because the US dollar is a dominant trading currency for oil, this petrol-dollar recycling has brought significant energy transaction costs to China. Thus, China should distribute energy trading risks across multiple currency types with financial instruments to hedge certain currency exposures. Despite Iran’s institutional isolation, Iran agreed to settle the oil trade in RMB, including China’s latest digital currency. This could protect China from devaluing the US dollar by using RMB to pay oil suppliers. Leverage decentralization and powerful encryption of block-chain technology can improve energy security and resilience. By unifying energy trading standards, China should consider to build a novel and interconnected energy trading platform with the use of multiple currency types.

## Conclusion and suggestion

In summary, this study focuses on how to protect China’s energy security in the wake of the recent China-US trade dispute and the COVID-19 pandemic. As China’s merchandise surplus and international trade have decreased since 2018, its foreign exchange reserves for energy trade will be insufficient. In China, an energy shortage will lead to higher prices and even a crisis if energy imports appear to dry up. Because China’s export-driven manufacturing industry is sensitive to energy prices, rising energy costs will raise manufacturing costs and weaken its competitiveness. China’s energy requirements will decrease; however, its energy supply vulnerability will increase. In addition, the long-term trade dispute would lead to a serious decline in China’s total external trade, reduced trade surplus and pressure on energy imports. As a result, overdue loans, real estate collapses, a food crisis and an energy crisis would occur because of the trade dispute and COVID-19 pandemic. To face these challenges, China should actively adopt a less externally dependent energy policy to ensure the security and sustainability of energy supply and maintain reasonable diplomatic relationships with major energy suppliers. Based on these, we recommend the following measures.

I. Construct cleaner power generation systems to meet environmental protection and emission requirements. China should increase investments in product innovation in renewable energy production systems to meet environmental and emissions requirements. Its transition to “carbon neutrality” calls for a comprehensive and systemic transformation of the economy. The promotion of the use of natural gas, solar, wind and other low-carbon power plants with the help of government rebates and subsidies is conducive to reducing energy supply risks.
II. Speed up advances in shale gas and oil technology with better use of China’s abundant coal gas resources. Developing shale gas and oil will be of significant benefits to China in the regions of the South China Sea, the East China Sea, the Sichuan Basin, and the Tarim Basin. Basically, China wants to make sure domestic pipelines are within its dominant application scope. China should speed up the development of unconventional natural gas technology, including downhole water separation, hydrogen sulphide removal, and deep-water robots.
III. Sponsor renewable energy vehicles and relevant energy-efficient and energy-cleaner technology. The completely electric vehicles, represented by Tesla, have expanded on family cars and freight trucks, covering a complete category of automobiles. Transport and charging networks for electric vehicles, as well as energy storage facilities with ultra-fast storage and instant charging devices, would become the standard energy infrastructure. The breakthrough of new energy storage technologies like the graphene battery and the ultra-capacitor will speed up the process.
IV. Update energy transactions and regulations through block-chain digital currency. Shortly, China will have to establish virtual currency and digital wallets at the national level. Some of China’s foreign exchange reserves and exported-oriented industrial capitals may be used as the endorsement for virtual currency for energy transactions and settlements. Eventually, the aim is to establish a faster, safer, and more distributed energy trading financial system, jumping out of the cycle of the “Dollar-Oil” financial system. These new decentralized energy deals and agreements are essential to China’s future energy security.
V. Engage in more aggressive energy diplomacy. China should continue to seek strategic energy partners as new suppliers via the “One-belt One-road” initiative, by increasing its investment in Russia, Central Asian countries (e.g., Kazakhstan), Middle Eastern countries (e.g., Iran, Iraq, UAE and Saudi Arabia) and South American countries (e.g., Brazil) to enhance its economic outlook and energy security. To develop a safer and more sustainable energy supply network, China should continuously provide financial aid and business cooperation in these resource-rich countries.

Regarding future research directions, we will extend this work to consider the iron ore supply chain, especially using the public data of the Australian mining industry [57]. Besides, we will implement operations research (i.e., Mixed Integer Programming), machine learning and game theory techniques as analytical tools to deal with quantitative energy security management problems [58–61].

## Data Availability

The datasets generated and/or analysed during the current study are accessible in the website of China’s National Bureau of Statistics (http://www.stats.gov.cn/english). Most of datasets generated and/or analysed during this study are included in Tables 1, 2, 3, 4, 6 and 7 of this published article. The datasets generated and/or analysed during the current study are available from the corresponding author upon reasonable requests.
